# The dynamics of self-control: within-participant modeling of binary food choices and underlying decision processes as a function of restrained eating

**DOI:** 10.1007/s00426-019-01185-3

**Published:** 2019-04-19

**Authors:** Claudio Georgii, Michael Schulte-Mecklenbeck, Anna Richard, Zoé Van Dyck, Jens Blechert

**Affiliations:** 1 Department of Psychology, Centre for Cognitive Neuroscience, Paris-Lodron-University of Salzburg, Hellbrunner Straße 34, 5020 Salzburg, Austria; 2 Institute of Marketing and Management, University of Bern, Bern, Switzerland; 3 Max Planck Institute for Human Development, Berlin, Germany; 4 Schoen Clinic Roseneck, Prien, Germany; 5 Faculty of Language and Literature, Humanities, Arts and Education, University of Luxembourg, Luxembourg City, Luxembourg

## Abstract

Successful self-control during food choice might require inhibition of impulses to avoid indulging in tempting but calorie-dense foods, and this might particularly apply to individuals restraining their food intake. Adopting a novel within-participant modeling approach, we tested 62 females during a mouse-tracking based binary food choice task. Subsequent ratings of foods on palatability, healthiness, and calorie density were modeled as predictors for both decision outcome (choice) and decision process (measures of self-control conflict) while considering the moderating role of restrained eating. Results revealed that individuals higher on restrained eating were less likely to choose more high-calorie foods and showed less self-control conflict when choosing healthier foods. The latter finding is in contrast with the common assumption of self-control as requiring effortful and conscious inhibition of temptation impulses. Interestingly, restrained eaters rated healthy and low-calorie foods as more palatable than individuals with lower restrained eating scores, both in the main experiment and an independent replication study, hinting at an automatic and rather effortless mechanism of self-control (palatability shift) that obviates effortful inhibition of temptation impulses.

**Electronic supplementary material:**

The online version of this article (10.1007/s00426-019-01185-3) contains supplementary material, which is available to authorized users.

## Introduction

Consumers face a daily struggle between maintaining a healthy eating style propagated by nutritionists and medical experts, and giving into immediate food temptations. Resisting tempting but energy dense foods is considered to require constant successful self-control (Baumeister, Vohs, & Tice, 2007; Hofmann, Friese, & Strack, 2009), defined as a preference for larger, but delayed, rewards (e.g., weight loss) over smaller, but immediate, rewards (e.g., eating chocolate; Mischel, Shoda, & Rodriguez, 1989). However, the pandemic rates of overweight and obesity (Haftenberger et al., 2016; Schienkiewitz, Mensink, Kuhnert, & Lange, 2017) and the low long-term success of weight-loss diets (Mann et al., 2007) indicate that self-control is prone to failures.

In search of the mechanisms underlying this varying success in self-control, one eating style has been studied intensely: *Restrained eating* describes a pattern of restricted food intake and weight watching to reduce or maintain weight (Schaumberg, Anderson, Anderson, Reilly, & Gorrell, 2016). Yet, the literature is mixed as to whether restrained eaters are actually successful in cutting down on intake: Laboratory food intake is often reduced in restrained eaters (Robinson et al., 2017). However, in several studies in naturalistic settings, psychometric measures of restrained eating do not consistently relate to actual calorie intake (e.g., Stice, Cooper, Schoeller, Tappe, & Lowe, 2007; Stice, Fisher, & Lowe, 2004; Stice, Sysko, Roberto, & Allison, 2010). Furthermore, a hallmark finding is that restrained eaters overeat after a perceived breach of their diet (Herman & Polivy, 1984).

One prominent theoretical explanation of why restrained eaters may be unsuccessful in exerting self-control in eating—despite their explicit intention to do so—is the need for *effortful and conscious inhibition* of temptation impulses (for a critical overview see Fujita, 2011). This approach states that in order to avert self-control failures, tempting impulses need to be consciously recognized as undesirable and then need to be inhibited. Thus, self-control failures occur due to the inability to inhibit such impulses, e.g., due to depleted cognitive resources, reduced motivation to exert self-control/attention to gratification (Inzlicht & Schmeichel, 2012; Inzlicht, Schmeichel, & Macrae, 2014) or particularly strong temptations (Kotabe & Hofmann, 2015; Stroebe, Mensink, Aarts, Schut, & Kruglanski, 2008). However, as proposed by Fujita (2011), viewing effortful impulse inhibition as the defining criteria for self-control neglects people’s capacity to monitor and process environmental information in a cognitively efficient way. The routinization and automatization of goal-striving behaviors, which would be less resource-demanding, would enable restrained eaters to enact self-control *without effortful and conscious* inhibition of temptation impulses (Bargh & Chartrand, 1999; Fishbach, Friedman, & Kruglanski, 2003; Papies, Stroebe, & Aarts, 2008). Taken together, it remains unclear whether restrained eaters do actually reduce food intake in line with their intentions and which type of self-control processes (conscious/effortful vs. non-conscious/effortless) are enacted to produce goal-consistent behavior.

Food choice is central to successful self-control but represents a rather challenging task: Average grocery stores host thousands of products, so how does one choose the small number of foods needed, when choices are affected by several, potentially conflicting, motivational dimensions? Besides economic, ethical, and cultural reasons, food choices are determined by palatability, calorie density, and healthiness (Köster, 2009; Leng et al., 2016; Mela, 2001; Steptoe, Pollard, & Wardle, 1995), the latter three being most relevant for weight- and self-control and often in conflict. For example, van der Laan, de Ridder, Charbonnier, Viergever and Smeets (2014) contrasted one condition with a maximized choice conflict between palatability and calorie density and another condition without this conflict, to examine the need to exert self-control through inhibition of temptation impulses. Surprisingly, in the self-control condition, in which palatability had to be discounted to choose low energy foods, weight-concerned women showed less experienced conflict (shorter reaction times and decreased brain activity in conflict monitoring regions). The authors concluded that effortful inhibition of temptation impulses is absent in their sample of weight-concerned women, possibly because their weight-control goals and respective self-control processes were not activated. Yet, even in weight-concerned individuals with high levels of (self-reported) self-control, Stillman, Medvedev, and Ferguson (2017) did not find an indication of effortful inhibition of temptation impulses that may arise from a conflict between food-enjoyment goals and weight-watching goals. This raises the question of whether less experienced conflict during food choice indicates an absence of self-control through effortful and conscious inhibition of temptation impulses or points to less resource-demanding mechanisms of self-control without conscious deliberation.

Distinguishing between these two types of self-control would require a measure of the effort that needs to be invested in aligning one’s behavior with overarching goals (e.g., weight reduction) in the face of several, potentially conflicting motivations. One promising methodological approach to measure continuous competition between various motivational forces during binary choice is afforded by the mouse-tracking technique (Freeman & Ambady, 2010; Freeman, Dale, & Farmer, 2011; Stillman, Shen, & Ferguson, 2018; Sullivan, Hutcherson, Harris, & Rangel, 2015). In contrast to traditional self-report-based metrics, which are prone to memory and other biases (Gorin & Stone, 2001) and metrics as reaction time (Stillman et al., 2017), it is assumed that mouse-tracking continuously measures real-time motor traces of cognitive processes and that less direct mouse traces toward a preferred choice option is indicative of a stronger underlying motivational conflict. Thus, mouse trajectories allow a deeper understanding of how different types of self-control facilitate healthy food choices (Lopez, Stillman, Heatherton, & Freeman, 2018).

One methodological constraint of most food choice tasks is that the expected self-control conflict has to be modeled a priori: For example, trials are artificially constructed for each participant—by selecting pre-rated food images (e.g., high palatable vs. low caloric)—to induce self-control conflicts (e.g., van der Laan et al., 2014). As other researchers have argued, the a priori construction of food pairs limits the generalizability to real-world decisions (e.g., Lopez et al., 2018). Thus, the present study took a novel approach to this methodological problem by realizing all possible food pairings of a representative set of foods during binary choice. Mixed-effects modeling was used to better characterize participants’ trial-level choice behaviors as a function of both trial-level features (subjective ratings on important choice dimensions as: palatability, health, calorie density) and person-level characteristics (i.e., restrained eating).

Using this approach, we hypothesized that choice would be primarily predicted by palatability preferences but—secondly—also by calorie density and perceived healthiness of the two food options (Raghunathan, Walker Naylor, & Hoyer, 2006; van der Laan et al., 2014), and that the latter two dimensions would be more influential in restrained eaters. Due to the inconsistent literature on food intake—as reviewed above—we did not make directional predictions as to whether restrained eaters would choose foods with lower or higher caloric density. Beyond choice behavior, we aimed to determine the *types of self*-*control* underlying food choice in restrained eaters: An conscious and effortful type of self-control would predict more conflict in restrained eaters, as manifested in less direct mouse trajectories, whereas a less conscious and effortful mechanism would predict the opposite. Due to these two contrasting theoretical accounts regarding self-control type, we anticipated additional exploratory analyses.

## Methods

### Participants

Sixty-nine female participants were recruited at the University of Salzburg, Austria. Due to non-compliance (i.e., not adhering to the study protocol) and technical issues, seven subjects were excluded from analyses, leaving 62 participants for the final analysis. Exclusion criteria were (a) current/past eating disorders, (b) current/past mental or neurological disorders, (c) vegan/vegetarian diet, and (d) food allergies (a–d assessed by written self-report). The study was approved by the University’s ethics committee and all experiments were performed in accordance with relevant guidelines and regulations. Informed consent form was obtained by all participants and signed by adult participants or the parents of underage participants (*n *= 3). Participants received course credit or a payment of €55. Average age was 22.2 years (SD = 3.98, range 16–35) and average body mass index was 22.2 kg/m^2^ (SD = 3.11, range 16.2–33.0). Restrained eating was measured with the Dutch Eating Behaviour Questionnaire (Van Strien, Frijters, Bergers, & Defares, 1986; *M* = 25.0, SD = 8.11, range 11–41).

### Procedure

Prior to the laboratory session, participants completed a set of questionnaires including the restrained eating subscale of the Dutch Eating Behaviour Questionnaire (10 items, e.g., “Do you take into account your weight with what you eat?”; Cronbach’s *α* = .888). To limit variability on hunger, participants were instructed to consume one out of five preset lunches (~ 550 kcal) 3 h prior to testing. Laboratory testing commenced with the attachment of sensors for physiological measurements (i.e., EEG, respiration, heart rate; data are not reported here). The food choice task started after resting baselines (~ 10 min) and a ~ 40 min emotional eating task (see Blechert, Goltsche, Herbert, & Wilhelm, 2014 for a similar task) assessing food cue responding under neutral and negative emotional state (order counterbalanced across participants).

#### Food choice task

**Fig. 1:**
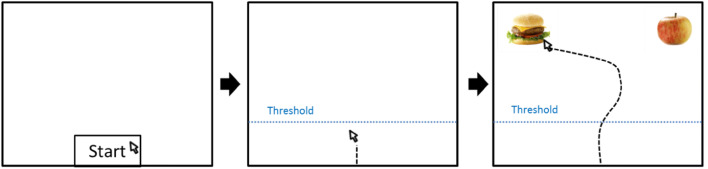
Example of a trial during the food choice task. All three boxes represent different stages of the trial in chronological order. Appearance of the two food pictures is triggered by the mouse cursor crossing the threshold (horizontal dotted line in middle and right panel, invisible to the participant). Food pictures displayed in this figure are derived from the food-pics database (URL: Food-pics) and reused under a Creative Commons License

To render the food choice task naturalistic, participants were instructed to select the one out of two food options that they would prefer to eat later, and that the five most frequently chosen foods would be available to them for tasting/eating after the task (in fact, all foods were available). The food choice task was presented using E-Prime 2.0 (Psychology Software Tools, Inc., Pittsburgh, PA, USA). On each trial (Fig. 1), participants, when they felt ready to start the trial, clicked on a small rectangle labeled “Start” at the bottom center of the screen and were instructed to move the mouse continuously to the upper part of the screen. After crossing a threshold (10% of the vertical screen resolution), two food pictures appeared, one in the upper-left and one in the upper-right corner of the screen. The trial ended with participants’ choice for one food or with exceeding the maximum trial duration of 4000 ms after the picture onset. To realize all possible combinations between the 18 foods^1^ (see online supplementary material figure S1), 153 trials were presented to the participant in individually randomized order (approximate duration of the task was 15 min).

#### Card rating task

After the choice task, each food was rated by ‘sorting’ printed food image cards along a Visual Analogue Scale (VAS; ranging from 0 to 100; 50 cm) separately for the motivational dimensions “momentary desire to eat” (“Please sort the foods by your desire to eat them right now”), “palatability” (“…by your general liking”), “calories” (“…by their calorie content”) and “health” (“…by their healthiness”). The order of motive dimension was random for each participant and the food cards were re-shuffled for each dimension (for an illustrative figure, see supplement S2).

#### Actual food intake

After the food rating task, all 18 foods were prepared and presented to the participant. Participants were free to choose which foods to taste and gave taste ratings (cover-story). Experimenters mentioned that foods had to be disposed afterwards and left the room for a more private, temporally unlimited eating situation. Unbeknownst to the participants, pre- to post-taste test weight of each food was later measured to the nearest gram and expressed in proportion of offered amount (i.e., consumed grams divided by available grams).

### Data analyses

#### Mixed-effects modeling strategy

R (R Core Team, 2016) and the R package *lme4* (Bates, Maechler, Bolker, & Walker, 2015) were used to calculate linear mixed-effects models. Generally, Level 1 represented trials, which were nested within participants, modeled on Level 2. To select an optimal fixed effects and random effects structure, we followed a stepwise, top-down model-selection strategy (Diggle, Heagerty, Liang, & Zeger, 2002; Zuur, Ieno, Walker, Saveliev, & Smith, 2009): Firstly, a ‘*beyond optimal model’* was calculated, including all theoretically interesting main and interactions as fixed effects. Secondly, *random models* tested whether modeling the various predictor slopes as random improved the overall model fit on Akaike Information Criterion (AIC) and Bayesian Information Criterion (BIC) to find the optimal random effects structure. Thirdly, we removed those predictors from the *random model* in a stepwise, backward deletion strategy, which did not lead to a significant reduction in AIC/BIC. The remaining *winning model* was then calculated using restricted maximum-likelihood-estimation. Generally, all predictors were z-standardized using the person-mean (Level 1) or the grand-mean (Level 2; restrained eating). All plots and tables were generated using *sjPlot* (Lüdecke, 2017); observed power of (significant) fixed effects was calculated using *simr* (Green & MacLeod, 2016). Exact model specifications (e.g., random slopes, distributions, power) differed across analyses and are thus described together with the respective results. Analyses modeled main effects of the covariates age, body mass index, and whether the participant ended the emotional eating task in the neutral or negative emotional condition (termed CondFirst). The latter factor was included in the analysis to assess potential carry-over effects into the present task, for example, that residual negative emotions would modulate impulse strength or neural reward processing (and do so differentially in restrained eaters, e.g., Wagner, Boswell, Kelley, & Heatherton, 2012). Covariates that neither yielded significant main effects nor altered the general pattern of results were not included in the winning model.

#### Analysis of mouse trajectories

As suggested by previous research (Freeman et al., 2011; Stillman et al., 2017), the trajectory of the mouse is influenced by the ongoing decisional process, and metrics derived from it can capture conflict based on underlying self-control processes. One conflict metric is the area under the curve (AUC) which reflects the degree of deviation from an ideal trajectory (equivalent to a straight line) to the selected option.

The AUC for each mouse trajectory was computed by

$${\text{AUC}} = n^{ - 1} \mathop \sum \limits_{i = 0}^{n - 1} a\cos \left( {\frac{{\overset{\lower0.5em\hbox{$\smash{\scriptscriptstyle\rightharpoonup}$}} {d}_{i} * \overset{\lower0.5em\hbox{$\smash{\scriptscriptstyle\rightharpoonup}$}} {t}_{i} }}{{\left| {\overset{\lower0.5em\hbox{$\smash{\scriptscriptstyle\rightharpoonup}$}} {d}_{i} } \right|* \left| {\overset{\lower0.5em\hbox{$\smash{\scriptscriptstyle\rightharpoonup}$}} {t}_{i} } \right|}}} \right),$$

whereas *n* denotes the number of elements in the vector, the vector $$\overset{\lower0.5em\hbox{$\smash{\scriptscriptstyle\rightharpoonup}$}} {d}$$ is defined by

$$\overset{\lower0.5em\hbox{$\smash{\scriptscriptstyle\rightharpoonup}$}} {d}_{i - 1} = \overset{\lower0.5em\hbox{$\smash{\scriptscriptstyle\rightharpoonup}$}} {p}_{i} - \overset{\lower0.5em\hbox{$\smash{\scriptscriptstyle\rightharpoonup}$}} {p}_{i - 1} .$$

And the vector $$\overset{\lower0.5em\hbox{$\smash{\scriptscriptstyle\rightharpoonup}$}} {t}$$ is defined by

$$\overset{\lower0.5em\hbox{$\smash{\scriptscriptstyle\rightharpoonup}$}} {t}_{i - 1} = \overset{\lower0.5em\hbox{$\smash{\scriptscriptstyle\rightharpoonup}$}} {a} - \overset{\lower0.5em\hbox{$\smash{\scriptscriptstyle\rightharpoonup}$}} {p}_{i - 1} ,$$

with *i* > 1, $$\overset{\lower0.5em\hbox{$\smash{\scriptscriptstyle\rightharpoonup}$}} {p}$$ is the path-vector (*x*- and *y*-coordinate of each measurement point) and $$\overset{\lower0.5em\hbox{$\smash{\scriptscriptstyle\rightharpoonup}$}} {a}$$ is the target vector (*x*- and *y*-coordinate of the target).

Another validated conflict metric is the number of *x*-*flips* (Freeman et al., 2011), which reflects the directional changes along the *x*-axis.

#### Data availability

The datasets generated during and/or analysed during the current study as well as the stimulus material and the experiment are available in the OSF repository, Link to OSF

#### Code availability

The custom R code to calculate both the *x*-flips and AUC metric is shared on a Github repository (Link to Github repository).

## Results

### Validation of the food choice task

**Fig. 2:**
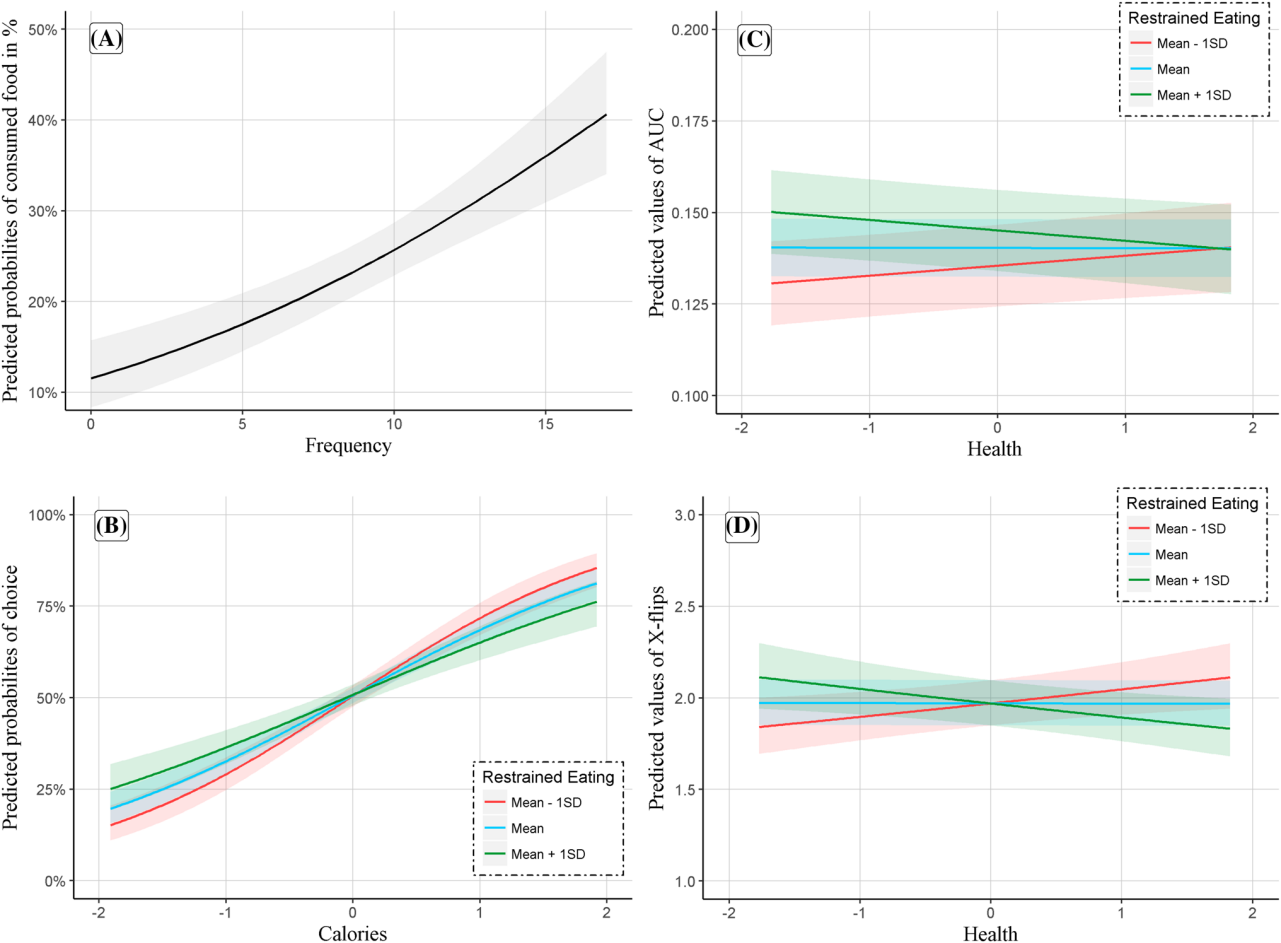
Validation of binary choice in the food choice task against actual food intake at the taste test (**a**) as well as modulatory role of restrained eating on the influence of calorie density/healthiness on food choice (**b**), area under the curve, AUC (**c**), and x-flips (**d**)

Are the serial food choices predictive of actual consumption? To determine this, we predicted the amount actually eaten (calculated as proportion to the amount offered) from frequency of choice in the food choice task. Results revealed that number of choices for a given food positively predicted the amount consumed (OR = 1.10, *p* < .001), indicating criterion/external validity for the food choice task (Fig. 2a).

### Analysis of choice as a function of motives and restrained eating

Which role does each motivational dimension play in food choice and is this role influenced by restrained eating? To evaluate this question, we subtracted motive strength of the non-selected from the selected food on each motive dimension, resulting in three difference scores (∆ health, ∆ palatability, ∆ calories). For example, a ∆ health score of 50 would reflect a 50-point advantage of the chosen vs. the non-chosen option. We calculated the motive difference scores for each of the 153 food picture pairings during the choice task. Across all 153 trials, binary choice (1-chosen, 0-non-chosen, assuming a binomial distribution) was predicted by each of the motive difference scores (on trial level/Level 1) along with their respective interactions with restrained eating as predictors in the *beyond optimal model*. AIC comparisons determined health as the best random slope for the *random model* (left column of Table 1) and *winning model* (right column of Table 1).

**Table 1:** Odds ratios (OR), their confidence intervals (CI), the standard errors (SE), power and *p* values for the mixed model with the predictors health, calories, palatability, restrained eating and their cross-level interactions on Choice

	OR	Random model				CI	Winning model		
		CI	SE	*p*	OR		SE	*p*	Power
Fixed parts									
(Intercept)	0.99	[0.88; 1.12]	0.06	.878	1.04	[0.96; 1.13]	0.04	.378	
Age	1.04	[0.95; 1.14]	0.05	.359					
Body mass index	0.98	[0.84; 1.13]	0.08	.743					
CondFirst	1.08	[0.90; 1.30]	0.10	.425					
Restrained eating	1.02	[0.92; 1.13]	0.05	.683	1.01	[0.93; 1.09]	0.04	.886	
Health	1.21	[0.97; 1.51]	0.11	.098	1.23	[0.98; 1.53]	0.14	.070	
Calories	2.06	[1.80; 2.35]	0.07	**< .001**	2.12	[1.86; 2.43]	0.15	**< .001**	100.00% [99.63; 100.00]
Palatability	9.16	[8.31; 10.09]	0.05	**< .001**	9.58	[8.67; 10.58]	0.49	**< .001**	100.00% [99.63; 100.00]
Health * restrained eating	1.01	[0.81; 1.27]	0.12	.928					
Calories * restrained eating	0.87	[0.76; 0.99]	0.07	**.038**	0.85	[0.76; 0.95]	0.05	**.004**	81.10% [78.53; 83.48]
Palatability * restrained eating	0.92	[0.83; 1.01]	0.05	.074					
Random parts									
*τ*_00, Participant_	0.058				0.058				
*τ*_01, Health_	0.490				0.491				
*N*_Participant_	62				62				
Observations	9317				9317				
AIC	7780.718				7713.799				
Hosmer–Lemeshow–*Χ*^2^	5.518; *p *= .701				7.894; *p *= .444				

Foods that were generally liked more (*z* value increase of 1) were nine times more likely to be chosen. Yet, individuals with higher levels of restrained eating were relatively less likely to choose a more energy dense food (Fig. 2b) than individuals with lower levels of restrained eating.^2^

### Measures of conflict in the food choice task: AUC and *x*-flips

If restrained eaters are successful in their choice behaviour, do they need to exert effortful and conscious self-control as indicated by increases in measures of conflict? We used the above model structure (motive difference scores as predictors) to predict AUC (Gaussian distribution) and *x*-flips (Poisson distribution) during choice process. The best *random model* for AUC (Table 2) determined health as the random slope and the best *random model* for *x*-flips (Table 3) was a random intercept model.

**Table 2:** Incidence rate ratios (IRR), their confidence intervals (CI), the standard errors (SE), power and *p* values for the mixed model with the predictors health, calories, palatability, restrained eating and their cross-level interactions on AUC

	Random model				Winning model				*Power*
	*B*	CI	SE	*p*	*B*	CI	SE	*p*	
Fixed parts									
(Intercept)	0.160	[0.151; 0.169]	0.006	**< .001**	0.140	[0.132; 0.148]	0.004	**< .001**	
Age	− 0.001	[− 0.008; 0.006]	0.004	.733					
Body mass index	0.000	[− 0.011; 0.011]	0.007	.973					
CondFirst	− 0.005	[− 0.019; 0.008]	0.008	.518					
Restrained eating	0.007	[− 0.001; 0.014]	0.005	.168	0.005	[− 0.003; 0.013]	0.004	.234	
Health	− 0.002	[− 0.004; 0.000]	0.001	.151					
Calories	− 0.004	[− 0.006; − 0.002]	0.001	**.001**	− 0.002	[− 0.003; − 0.001]	0.001	**< .001**	92.70% [90.91; 94.23]
Palatability	− 0.004	[− 0.005; − 0.004]	0.001	**< .001**	− 0.004	[− 0.005; − 0.003]	0.001	**< .001**	100.00% [99.63; 100.00]
Health * restrained eating	− 0.003	[− 0.005; − 0.001]	0.001	**.015**	− 0.003	[− 0.005; − 0.001]	0.001	**.016**	66.00% [62.97; 68.94]
Calories * restrained eating	− 0.002	[− 0.004; 0.000]	0.001	.064	− 0.002	[− 0.004; 0.000]	0.001	.072	
Palatability * restrained eating	0.000	[− 0.001; 0.002]	0.001	.621					
Random parts									
*σ*^2^	0.002				0.002				
*τ*_00, Participant_	0.001				0.001				
*τ*_01, Health_	0.527				0.525				
*N*_Participant_	62				62				
Observations	9317				9317				
AIC	− 28,313.829				− 28,289.893

**Table 3:** Standardized estimates (B), their confidence intervals (CI), the standard errors (SE), power and *p* values for the mixed model with the predictors health, calories, palatability, restrained eating and their cross-level interactions on X-flips

	Random model				Winning model				Power
	IRR	CI	SE	*p*	IRR	CI	SE	*p*	
Fixed parts									
(Intercept)	1.37	[1.27; 1.49]	0.04	**< .001**	1.97	[1.85; 2.10]	0.06	**< .001**	
Age	0.99	[0.93; 1.05]	0.03	.731					
Body mass index	0.99	[0.90; 1.09]	0.05	.787					
CondFirst	1.06	[0.93; 1.19]	0.06	.386					
Health	0.98	[0.95; 1.02]	0.02	.433					
Restrained eating	1.02	[0.96; 1.10]	0.03	.504					
Calories	0.96	[0.92; 1.00]	0.02	**.041**					
Palatability	0.94	[0.93; 0.96]	0.01	**< .001**	0.96	[0.95; 0.97]	0.01	**< .001**	99.90% [99.44; 100.00]
Health * restrained eating	0.96	[0.93; 1.00]	0.02	**.046**	0.96	[0.93; 0.99]	0.02	**.015**	64.60% [61.55; 67.57]
Calories * restrained eating	0.97	[0.94; 1.01]	0.02	.104	0.97	[0.94; 1.00]	0.02	**.038**	48.20% [45.06; 51.35]
Palatability * restrained eating	1.01	[0.99; 1.03]	0.01	.241					
Random parts									
*τ*_00, Participant_	0.060				0.060				
*N*_Participant_	62				62				
Observations	9317				9317				
AIC	30,706.238				30,701.300				
Deviance	8953.668				8957.670				

Results revealed that choices for more energy dense and generally more liked foods were characterized by a *smaller* AUC, demonstrating less conflict during choice. Crucially, though while health was no significant predictor on its own, individuals with higher restrained eating experienced less conflict during choices for healthier options, while the opposite was true for individuals with lower restrained eating (Fig. 2c).^3^ The pattern of results was similar for *x*-flips (Fig. 2d)^4^ such that individuals with higher restrained eating experienced less conflict, indicated by fewer x-flips, during choices for healthier options.

### Exploratory analysis: determinants of palatability

Restrained eaters showed healthier choices and did so without any indication of conflict (smaller AUCs and fewer *x*-flips). This result pattern motivated additional analyses: choice motives were investigated in more detail to determine whether restrained eaters had changed their palatability patterns in service of their dieting goal. Thus, palatability was predicted by the food motives calories and health in two separate analyses, each with restrained eating as moderator (cross-level interactions; Table 4).

**Table 4:** Standardized estimates (B), their confidence intervals (CI), the standard errors (SE), power and *p* values for the mixed models of the cross-level interaction between restrained eating with health and calories on palatability

	Palatability				Palatability				Power
	*B*	CI	SE	*p*	*B*	CI	SE	*p*	
Fixed parts									
(Intercept)	62.61	[60.46; 64.76]	1.31	**< .001**	62.61	[60.46; 64.76]	1.31	**< .001**	
Age	2.10	[0.43; 3.78]	1.02	**.043**	2.10	[0.43; 3.78]	1.02	**.043**	
Body mass index	1.02	[− 1.63; 3.67]	1.61	.530	1.02	[− 1.63; 3.67]	1.61	.530	
Restrained eating	0.56	[− 1.26; 2.38]	1.11	.615	0.56	[− 1.26; 2.38]	1.11	.615	
CondFirst	− 0.68	[− 3.99; 2.63]	2.01	.737	− 0.68	[− 3.99; 2.63]	2.01	.737	
Health	3.51	[1.86; 5.17]	1.01	**.001**					
Calories					− 0.50	[− 2.17; 1.17]	1.01	.621	
Health * restrained eating	3.80	[2.14; 5.46]	1.01	**< .001**					97.90% [96.81; 98.70]
Calories * restrained eating					− 3.36	[− 5.04; − 1.69]	1.02	**< .001**	90.90% [88.94; 92.61]
Random parts									
*σ*^2^	1033.569				1038.198				
*τ*_00, Participants_	6.427				6.845				
*N*_Participants_	62				62				
Observations	1116				1116				

Both columns display the respective winning model (optimal random and fixed structure)

**Fig. 3:**
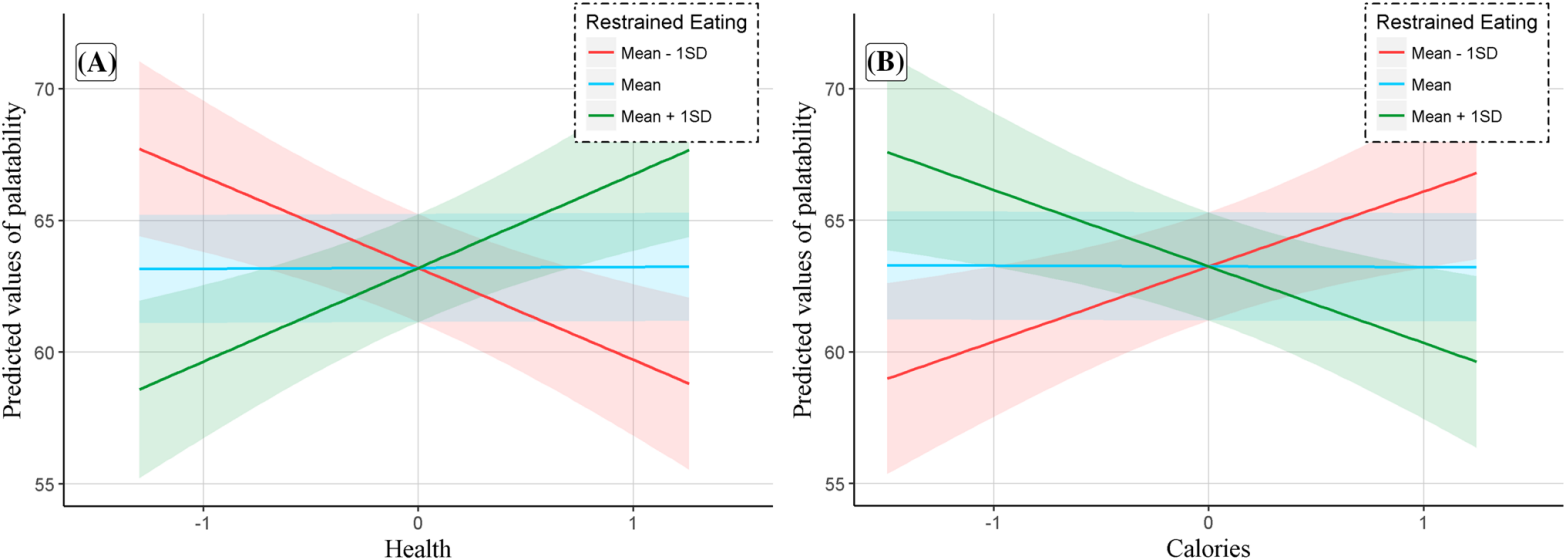
‘Palatability shift’: palatability increased as a function of health (**a**) and decreased as a function of calories (**b**) in restrained eaters while the reverse was true for unrestrained eaters

Results revealed that in individuals with higher levels of restrained eating rated healthier food options more palatable (Fig. 3a). The same modulatory pattern of restrained eating was observed with calories (Fig. 3b): in individuals with higher levels of restrained eating rated calorie-dense foods as less palatable.

### Follow-up study

Because the results obtained in the exploratory analysis were not planned a priori, we replicated them in an independent sample (*N* = 55) at three research centers (Salzburg, Austria; Goettingen, Germany; Nijmegen, the Netherlands). The card rating task was conducted with identical routines and materials. The results confirmed the palatability shift between restrained and unrestrained eaters found in the first study (see online supplement table S1).

## Discussion

The current study adopted a new experimental and statistical modeling approach to investigate determinants of food choice in restrained eating. The external validity of our new binary choice task was supported by strong and consistent associations of choice with actual food intake on a test meal. In line with our hypothesis and other research (e.g., Raghunathan et al., 2006), we showed that palatability is the main driver for food choice with health and calorie density having significant but subordinate roles. Furthermore, we are able to show that individuals with higher levels of restrained eating were less likely to choose highly palatable and calorie-dense foods than their counterparts with lower levels of restrained eating. Thus, restrained eaters’ choice pattern was in line with their weight-control goal.

Importantly, using the mouse-tracking technique we were able to investigate how restrained eaters executed such successful choices. According to accounts that equate self-control with *effortful and conscious inhibition of temptation impulses,* it would be expected that impulses associated with tempting foods are in conflict with restrained eaters’ health/weight goals. Such impulses would thus need to be inhibited—through slow and controlled processes—to support successful choice outcome. Yet, we found no indication of such effortful inhibition (or choice conflict) in our mouse-tracking data: instead individuals with higher levels of restrained eating demonstrated less conflict when choosing the healthier food option, as illustrated by fewer *x*-flips (direction reversals) and smaller AUCs (overall less strait decision path). This pattern bears similarity with the absence of choice conflict in weight-concerned individuals during binary food choice trials in the study by van der Laan et al. (2014). Such pattern could either be due to a reduced impulse strength (Hofmann et al., 2009; Kotabe & Hofmann, 2015), a lack of activation of weight-control goals (as hypothesized by van der Laan et al., 2014) or to the operation of non-conscious and effortless self-control mechanisms. In our search for reasons for the absence of decision conflicts in our data, we found that restrained eaters rated healthier and less energy dense foods as more palatable. Thus, we ran a second study to replicate this latter result in an independent sample, suggesting that the finding was not specific to our sample or that palatability ratings were not influenced by previous food choices (e.g., according to dissonance reduction; Izuma et al., 2010). Results across both studies showed that such an alteration or ‘shift’ in palatability preference from high palatable/caloric to healthy/less energy dense foods aligns restrained eaters’ food liking (or impulses) with their weight-control goal (similar results obtained by Buckland et al., 2015). Importantly, this ‘palatability shift’—potentially reflecting a more mid-to long-term attitude change—obviates the need for regulatory efforts to inhibit tempting impulses driven by attractive yet unhealthy foods.

More generally, as indicated above, much of eating behaviour research has explicitly or implicitly operated under the ‘effortful inhibition of impulses’ account. However, a simple equation of self-control with a slow, conscious, and effortful process has been repeatedly criticized (e.g., Fishbach et al., 2003; Fujita, 2011; Galla & Duckworth, 2015; Haynes, Kemps, & Moffitt, 2016; Neal, Wood, & Drolet, 2013). In fact, there has been growing awareness that information processing below the level of consciousness may have a stronger impact on choices and decision making than previously assumed (Bargh & Chartrand, 1999; Galla & Duckworth, 2015). In our view, the observed pattern of choice, process and rating data could be better contextualized within accounts that accommodate the operation of non-conscious and effortless types of self-control, reviewed and systematized in the dual motive framework by Fujita (2011). These accounts include goal priming, which refers to the establishment of facilitative links from temptations (high energy foods) to overarching goals through repeated successful goal pursuit (Fishbach et al., 2003). Relatedly, an initially effortful act (choosing a healthy instead of a palatable food) can become more efficient over time and practice until it proceeds without conscious guidance (Bargh & Chartrand, 1999; Hagger, Wood, Stiff, & Chatzisarantis, 2010), representing a process similar to skill acquisition or the development of habits (Verplanken, 2018). The palatability shift observed here could be related to either of these mechanisms, but may also constitute a strategy of its own. Future research could study such palatability changes longitudinally to determine when and how such changes take place.

These conclusions have to be seen in the light of some limitations. Generalization is limited to predominantly healthy-weight female individuals, given differences in health beliefs and dieting between women and men (e.g., Wardle et al., 2004) and between healthy and eating disordered samples (Foerde, Steinglass, Shohamy, & Walsh, 2015; Steinglass, Foerde, Kostro, Shohamy, & Walsh, 2015). Further, despite observing goal-consistent food choice behavior, we did not assess whether restrained eaters in this sample were actually successful in terms of every day dieting (see discussion around validity or restrained eating questionnaires; Ahern, Field, Yokum, Bohon, & Stice, 2010; Stice et al., 2004, 2007) or whether they had higher levels of self-control in general (as observed by Stillman et al., 2017). Thus, investigating objective dieting success (and maybe general self-control) in various populations with altered eating behavior would offer promising future directions.

To conclude, individuals with higher levels of restrained eating showed successful self-control in a binary food choice task, and they did so using a rather effortless and automatic mechanism, which might be related to a change in their palatability preferences (‘palatability shift’). This palatability change comprises the devaluation of temptation (i.e., less liking for more calorie-dense/unhealthy foods) as well as an increased valuation of goal-congruent foods (i.e., increased liking for less calorie-dense/healthy foods) and ultimately brings food preferences and long-term goals (i.e., weight reduction) into alignment. Such mechanisms would have been hard to detect without the current statistical modeling approach that employs individual image ratings as predictors of binary choice and associated process data. Thus, this approach might be applicable to other fields of decision-making research that study conflicts between multiple choice motives. Last, current weight loss treatments heavily emphasize effortful impulse inhibition, which might explain their vulnerability for failure during times of stress and limited cognitive control resources. To replace or at least complement these approaches, the discovery of ‘effortless’ mechanisms in food choice in the present study might fuel the development of corresponding interventions in more naturalistic dieting studies.

## Electronic supplementary material

Below is the link to the electronic supplementary material.

Supplementary material 1 (DOCX 330 kb)

## Footnotes

1 The pictures were taken from a database of standardized food images (Blechert, Meule, Busch, & Ohla, 2014).

2 Simple slope analyses revealed no significant slopes (*ps* > 0.05).

3 Simple slope analyses revealed significant slope only for high restrained eaters (> + 1SD; *z* = − 3.43, *p* <  0.001).

4 Simple slope analyses revealed significant slope only for high restrained eaters (> + 1SD; *z* = − 1.99, *p* = 0.046).
