# Supplementary material for: The dynamics of self-control: within-participant modeling of binary food choices and underlying decision processes as a function of restrained eating
Source: Psychol Res. 2019 Apr 19;84(7):1777–88. doi: 10.1007/s00426-019-01185-3 (PMC7478946; doi:10.1007/s00426-019-01185-3)
Supplement: Supplementary file 1 — Supplementary material 1 (DOCX 330 kb) [file 426_2019_1185_MOESM1_ESM.docx]

**Supplementary Material**

The dynamics of self-control: Within-participant modeling of binary food choices and underlying decision processes as a function of restrained eating

^­^Claudio Georgii^1,^*, Michael Schulte-Mecklenbeck^2,3^, Anna Richard^1,4^, Zoé Van Dyck^5^, & Jens Blechert^1^

^1^Paris-Lodron-University of Salzburg, Department of Psychology, Centre for Cognitive Neuroscience, Salzburg, Austria

^2^Institute of Marketing and Management, University of Bern, Bern, Switzerland

^3^Max Planck Institute for Human Development, Berlin, Germany

^4^Schoen Clinic Roseneck, Prien, Germany

^5^Faculty of Language and Literature, Humanities, Arts and Education, University of Luxembourg, Luxembourg

*Correspondence:

Claudio Georgii, MSc.

University of Salzburg, Department of Psychology


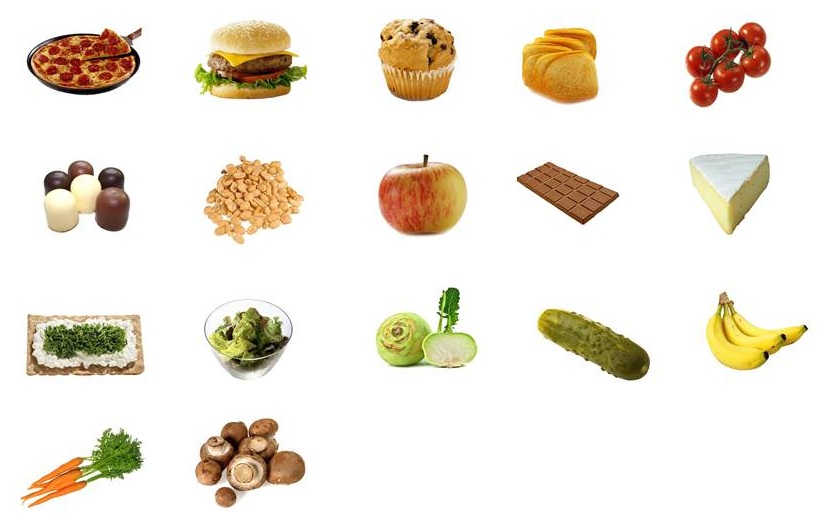


*Figure S1*. List of pictures retrieved from the food-pics database. The database is distributed under a Creative Common License ([URL: Food-pics](http://www.eat.sbg.ac.at/resources/food-pics)).

Table S1

*Standardized estimates (B), their confidence intervals (CI), the standard errors (SE), p-values for the mixed models of the cross-level interaction between restrained eating with health and calories on* ***Palatability****. Both columns display the respective Winning Model (optimal random and fixed structure).*

|  |  | Winning Model | | | |  | Winning Model | | | |
| --- | --- | --- | --- | --- | --- | --- | --- | --- | --- | --- |
|  |  | *B* | *CI* | *SE* | *p* |  | *B* | *CI* | *SE* | *p* |
| **Fixed Parts** | | | | | | | | | | |
| (Intercept) |  | 61.10 | [58.80; 63.39] | 1.17 | **<.001** |  | 61.20 | [58.93; 63.47] | 1.16 | **<.001** |
| Health*Restrained Eating |  | 1.92 | [-0.04; 3.88] | 1.00 | .055 |  |  |  |  |  |
| Calories*Restrained Eating |  |  |  |  |  |  | -2.27 | [-4.20; -0.35] | 0.98 | **.020** |
| **Random Parts** | | | | | | | | | | |
| σ^2^ |  | 982.749 | | | |  | 982.144 | | | |
| τ_00, VPN_ |  | 20.436 | | | |  | 18.910 | | | |
| N_VPN_ |  | 55 | | | |  | 55 | | | |
| Observations |  | 988 | | | |  | 988 | | | |

Table S2

*Descriptive statistics of all variables used in the analyses concerning the choice data.*

| *name* | *missing* | *complete* | *n* | *mean* | *sd* | *p0* | *p25* | *p50* | *p75* | *p100* |
| --- | --- | --- | --- | --- | --- | --- | --- | --- | --- | --- |
| VPN | 0 | 9317 | 9317 | 148.39 | 29.31 | 101 | 121 | 148 | 174 | 204 |
| Trial | 0 | 9317 | 9317 | 77.37 | 44.14 | 1 | 39 | 78 | 116 | 153 |
| RT | 0 | 9317 | 9317 | 1807.02 | 618.71 | 699 | 1351 | 1659 | 2122 | 3995 |
| AUC | 0 | 9317 | 9317 | 0.14 | 0.057 | 0.026 | 0.1 | 0.13 | 0.16 | 0.52 |
| Xflip | 0 | 9317 | 9317 | 2.03 | 1.39 | 0 | 1 | 2 | 3 | 10 |
| Palatability | 0 | 9317 | 9317 | 28.59 | 35.98 | -100 | 0 | 27 | 55 | 100 |
| Calories | 0 | 9317 | 9317 | 6.36 | 51.99 | -100 | -29 | 2 | 48 | 100 |
| Health | 0 | 9317 | 9317 | -1.8 | 55.73 | -100 | -45 | 0 | 36 | 100 |
| Restrained Eating | 0 | 9317 | 9317 | 24.93 | 8.05 | 11 | 18 | 25 | 33 | 41 |

Table S3

*Descriptive statistics of all variables used in the analyses concerning the rating data*

| *name* | *missing* | *complete* | *n* | *mean* | *sd* | *p0* | *p25* | *p50* | *p75* | *p100* |
| --- | --- | --- | --- | --- | --- | --- | --- | --- | --- | --- |
| VPN | 0 | 1054 | 1054 | 148.52 | 29.46 | 101 | 121 | 148.5 | 174 | 204 |
| Calories | 0 | 1054 | 1054 | 53.13 | 36.22 | 0 | 15 | 59.5 | 89 | 100 |
| Health | 0 | 1054 | 1054 | 52.02 | 38.82 | 0 | 10 | 54 | 95 | 100 |
| Liking | 0 | 1054 | 1054 | 62.2 | 32.14 | 0 | 40 | 70 | 94 | 100 |
| Frequency | 0 | 1054 | 1054 | 8.53 | 4.9 | 0 | 4 | 9 | 13 | 17 |
| Restrained Eating | 0 | 1054 | 1054 | 24.97 | 8.05 | 11 | 18 | 25 | 33 | 41 |


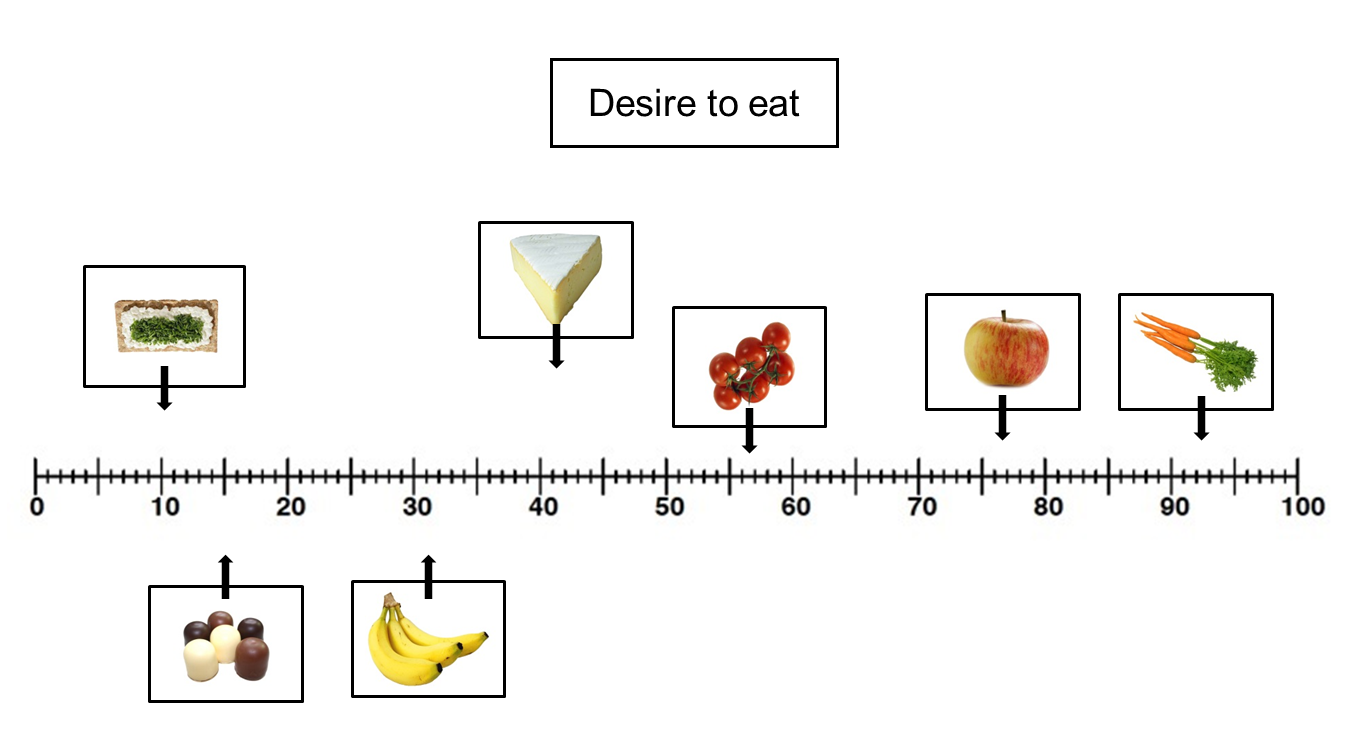


*Figure S2*. Illustrative “snapshot” of rating procedure using the Visual Analogue Scale. Food pictures displayed in this figure are derived from the food-pics database [(URL: Food-pics)](http://www.eat.sbg.ac.at/resources/food-pics) and reused under a Creative Commons License.
